# Supplementary material for: Content-rich biological network constructed by mining PubMed abstracts
Source: BMC Bioinformatics. 2004 Oct 8;5:147. doi: 10.1186/1471-2105-5-147 (PMC528731; doi:10.1186/1471-2105-5-147)
Supplement: Additional File 5 — The original Chilibot query results of the term "long-term potentiation (LTP)" and 22 other terms, limiting the latest references analyzed to the years 1990, 1995, 2000, and 2004. [file 1471-2105-5-147-S5.bz2 › chilibotAdditionalFile5/ltp2004/html/TRKA_PKA.html]

 


 **TRKA** and **PKA** 
  
Found 7 abstracts in PubMed,  **7 abstracts were retrieved and analyzed**.  


---

 Search Google  |
 PDF files only 
|  EDU domain only 

---

**Interactive relationship** (e.g. stimulation, inhibition, etc)

**Parallel relationship** (e.g. studied together, co-existance, homology, etc.)

- Because the RIbeta subunit is only present in the nervous system, including small diameter  **trkA**  receptor positive dorsal root ganglion cells, we suggest that in inflammatory conditions, RIbeta  **PKA**  is specifically required for nociceptive processing in the terminals of small diameter primary afferent fibers.  Ref: 9295392 J Neurosci, 1997
- Altogether, these results demonstrate that MC14 enhances the neurite outgrowth by cooperating at least two separated signaling pathways, a  **TrkA**  MAP kinases pathway and an adenylate cyclase  **PKA**  pathway, in PC12D cells.  Ref: 12850058 Int J Dev Neurosci< MedlineTA>Int J Dev Neurosci, 1997
- Moreover, simultaneous activation of the A R  **PKA**  CREB mediated and the phosphatidylinositol 3 kinase pathways caused neurite outgrowth that was not suppressed by a selective inhibitor of  **TrkA** , indicating that transactivation of  **TrkA**  was not involved.  Ref: 12114502 J Biol Chem, 2002
